# Transient Down-Regulation of Nucleoside Transporter 3 Gene Expression as a Drug Target in *Leishmania major Using* Antisense RNA Technology

**Published:** 2019

**Authors:** Farideh TOHIDI, Bahram KAZEMI, Mojgan BANDEHPOUR, Iraj SHARIFI, Mohammad Reza RABIEI, Ebrahim SAEDI DEZAKI, Zahra BABAEI

**Affiliations:** 1. Department of Medical Parasitology and Mycology, School of Medicine, Kerman University of Medical Sciences, Kerman, Iran; 2. Infectious Diseases Research Center, Golestan University of Medical Sciences, Gorgan, Iran; 3. Cellular and Molecular Biology Research Center, Shahid Beheshti University of Medical Sciences, Tehran, Iran; 4. Department of Biotechnology, School of Medicine, Shahid Beheshti University of Medical Sciences, Tehran, Iran; 5. Department of Biotechnology, School of Advanced Technologies in Medicine, Shahid Beheshti University of Medical Sciences, Tehran, Iran; 6. Leishmaniasis Research Center, Kerman University of Medical Sciences, Kerman, Iran; 7. Department of Statistics, School of Mathematical Sciences, Shahrood University of Technology, Shahrood, Iran; 8. Department of Medical Parasitology and Mycology, School of Medicine, Shahrekord University of Medical Sciences, Shahrekord, Iran

**Keywords:** Nucleoside transporter 3 gene, *Leishmania major*, Antisense RNA, Drug target, Gene expression

## Abstract

**Background:**

This study was aimed to silencing the Nucleoside transporter 3 (NT3) permease nucleobases involved in the salvage pathway of *Leishmania* in order to disrupt purine nucleotide uptake in the parasite and consequently, destruction of the parasite.

**Methods:**

Overall, 502 bp fragment of the NT3 gene sequence was designed to produce an antisense transcript upon entry of the vector into the parasite. The NT3 construct was transfected into *L. major* promastigotes and NT3 gene expression was studied in vivo and in vitro conditions.

**Results:**

Relative expression NT3 gene in transgenic *Leishmania* was decreased in tenth day. The percentages and the number of amastigotes infected macrophages with transgenic *L. major* were less than infected macrophages with wild-type strain. Our results in two groups of BALB/c female mice (wild-type strain and mutant, n=4 each group) were showed that size and number of ulcers in BALB/c mice infected with transgenic *Leishmania* promastigotes were less than the BALB/c mice infected with wild-type parasites.

**Conclusion:**

The results indicate the use of antisense RNA reduces of NT3 gene expression in *L. major*. More studies are required to obtain a new approach for treating *Leishmania* infection.

## Introduction

Leishmaniasis is a disease with a broad range of clinical manifestations caused by several species of the genus *Leishmania*, in the family *Trypanosomatidae* (1, 2). It is one of the most important parasitic diseases in 98 countries and manifests in cutaneous, visceral and cutaneous-mucosal forms (3). The life cycle of these parasites has two stages: amastigote that resides inside the reticuloendothelial cells of the vertebrate host and a slender, promastigote that replicates in medium and in the gut of the sandfly as a biological vector (1). Like all protozoa, *Leishmania* spp. are unable to synthesize cyclic purines *de novo* and must import purines from both their vertebrate and insect hosts (4, 5). In all organisms, purines are responsible for many vital functions, like the synthesis of DNA and RNA as well as precursors of coenzyme A, ATP, GTP, NADH, carbohydrates, lipids and the second messenger cAMP (6, 7). All parasite purine transporters are members of the Equilibrative Nucleoside Transporter SLC29 family in the human genome database (8, 9) or the 2.A57 family in the transporter classification database (10). Nucleoside transporters are present in mitochondria, cell membranes and lysosomes. *Leishmania* absorbs purines via purine permease through food and salvage. The first step of salvage is the displacement of purine nucleosides and nucleobases in the plasma membrane of the parasite. These hydrophilic nucleosides and nucleobases are unable to be released in the parasitic plasma membrane lipid layers, and proteins or transporters are needed to move them. The purine transporters are pharmacologically critical, as well as being important in human pathogenesis because they act as mediators in purine analogue uptake. Non-purine drugs such as allopurinol, which is an anti-*Leishmania* drug, are toxic to parasites when they enter into these organisms via purine transporters (4, 6, 7, 11, 12).

The *Leishmania* genome has four identified sites for purine transfer, known as nucleoside transporters 1–4 (NT1, NT2, NT3 and NT4) (6). *Leishmania major* expresses two purine nucleobase transporters, nucleoside transporters 3 and 4 (NT3 and NT4). Functional analysis of cloned transporter genes has revealed that purine nucleobases, including adenine, guanine, hypoxanthine and xanthine, are the specific targets of NT3 (6, 13, 14). NT3 permease expresses a large quantity of mRNA during the growth of parasites and has a high affinity for purine nucleobases. Although NT3 permease nucleobases are not essential for the parasite, NT3 gene deletion may reduce parasite viability and lead to the death of the parasite. These events underline the essential role of nucleobase transporters of *Leishmania* in purine uptake in both vertebrate and invertebrate hosts (14).

For this reason, this research was carried out to knocking-down the NT3 permease nucleobases that are involved in the salvage pathway of *Leishmania* in order to disrupt purine nucleotide uptake in the parasite, resulting in the death of the parasite.

## Materials and Methods

### NT3 plasmid construct

The 502 bp sequence (5′ end) of NT3 gene (Accession Number: XM_ 001681813) was considered as antisense. This antisense was synthesized by Generay Technology, Shanghai Co, China. Gene was subcloned into pcDNA 3.1 Neomycin vector by Hind III and EcoRI restriction enzymes. *E. coli* strain TOP 10 was selected and recombinant plasmid was transformed into it. Subsequently, plasmid was extracted and confirmed by Hind III and EcoRI restriction analysis. Presence of NT3 gene was confirmed by PCR method with following primers:

F: 5′- GGCGTACCTCTACTTCAGCC-3′, R: 5′- TTGATGGCACGGTACTCACC-3′.

### Culture of Leishmania major

*L. major* promastigotes (MRHO/IR/75/ER) were grown in complete DMEM culture medium containing 10% inactivated foetal bovine serum and 100U/ml penicillin and 100 μg/ml streptomycin and incubated at 25 °C. After 24–48 h, the cells were used for electroporation (15).

### Transfection

*L. major* promastigotes at stationary phase with concentration of 1 × 10^6^/ml were washed in PBS buffer and resuspended in electroporation buffer (20mM HEPES, pH 7.2, 137mM NaCl, 5 mM KCl, 0.7 mM Na_2_HPO_4_, and 6mM Glucose). Overall, 350 μl promastigotes were transferred to a 0.2 cm electroporation cuvette (Eppendorf, Germany) containing 2 ng/μl plasmid DNA harbouring the antisense NT3 gene. Electroporation was performed at 1000 V with a capacitance of 160 μs. Transfected cells were incubated on ice for 10 min, then transferred to 2 ml complete DMEM medium without antibiotics, and finally incubated at 25 °C for 20–24 h. On the second day, after centrifugation of cell cultures and removal of the supernatant, the precipitate was dissolved in the suspension obtained and transferred into 1cc complete DMEM medium containing 50 μg/ml G418 antibiotics (Sigma, USA) and incubated at 25 °C. This culture medium was checked every day for cell number, morphology and motility under a microscope (15–17). Wild-type *L. major* as positive control was electroporated.

### Quantitative real time-RT PCR

Total RNA was extracted from the mutant and wild-type promastigotes on 3, 7, 10, 15 and 20 d after transfection by total RNA Purification kit (Jena Bioscience, Germany). The cDNA synthesized was performed using AccuPower RT PreMix (Bioneer, Korea). The rRNA 45 gene of *L. major* was used as a housekeeping gene (18). The following PCR cycling conditions: denaturation 55 °C for 30 sec; amplification for 12 cycles of 20 °C (30 sec), 42 °C (4 min) and 95 °C (5 min). Real-time PCR was performed with 1 μl of cDNA, 2 μl of 5 pmol primers and 5 μl 2 × Greenstar qPCR Master Mix (Bioneer, Korea) and 2 μl of deionized water in a final volume of 10 μl. The real-time PCR program in the Rotor gene 6000 (Qiagen, USA) was as follows: 94 °C for 2 min and 94 °C for 20 sec; 40 cycles of 53 °C for 20 sec and 72 °C for 30 sec. Relative quantity (RQ) values were calculated using the 2^−ΔΔCt^ method.

### Western blot analysis

Pellets of mutant and wild-type *Leishmania* promastigotes (after transfection) were collected by centrifugation on day 3, 7, 10, 15 and 20; proteins were extracted into lysis buffer and separated by SDS-PAGE in 10% acrylamide gel (19). The protein bands were transferred to a nitrocellulose membrane. NT3-specific sheep antibody was used as a primary antibody with titer of 1:1000 (Genscript, USA). Rabbit anti-sheep IgG antibody conjugated with alkaline phosphatase (as a 1:10000 dilution) was used for the detection of NT3 protein.

### Macrophage infection assay

The murine macrophages cell line J774 were cultured at a concentration of 2 × 10^5^ in RPMI-1640 containing 10% inactivated FBS, 100 U/ml penicillin and 100 μg/ml streptomycin in Nunc 24-well plates. These plates were incubated at 37 °C with 5% CO_2_ (20). After five h, the culture medium was changed. Then, 24 h later, 1 × 10^6^ transgenic *L. major* promastigotes and wild-type *L. major* promastigotes were added into the Nunc^®^ 24-well plates containing cell line J774. The plates were incubated at 37 °C with 5% CO_2_ (20). Two smears were prepared from the plates after 24, 48 and 72 h and Giemsa staining were performed. In each smear, the percentage of infected macrophages and the mean number of amastigotes per infected macrophages in one hundred macrophages were calculated. The number of amastigotes in macrophages and the percentage of infected macrophages were counted.

### Infectivity in BALB/c mice

Two groups (n = 4) of BALB/c female mice (4–6 wk of age), 20–25 gr and were used. Mice of one group were infected with wild-type and another group were infected with mutant promastigotes. Mice for macroscopic examination with respect to ulcer(s) incubation period; count and diameter form each group was studied. Wild-type and transgenic promastigotes a concentration of 1.5 × 10^8^ (21) were injected subcutaneously into the base of the tails of mice in a 0.1 ml volume (22). The mice were examined macroscopically for 4 wk after infection.

### Ethics Statements

The Ethics Committee on Laboratory Animals at Kerman University of Medical Sciences (KMU) approved the study protocols of this project (Code of Number ethics: 92/78, 2013). This research was performed in compliance with the recommendations and guidelines for the use and surveillance of laboratory animals of International Institute. Nevertheless, all efforts were made to minimize animal suffering.

### Statistical analysis

Data analysis was performed using SPSS version 22.0 (SPSS Inc., Chicago, IL, USA) and ANOVA was used for statistically significant differences (*P*<0.05).

## Results

NT3 fragment antisense was cloned in pcDNA 3.1 Neomycin+ vector. By using NT3-specific primers, PCR was performed and the 500-bp cloned fragment was isolated.

### NT3 gene expression

**Fig. 1:**
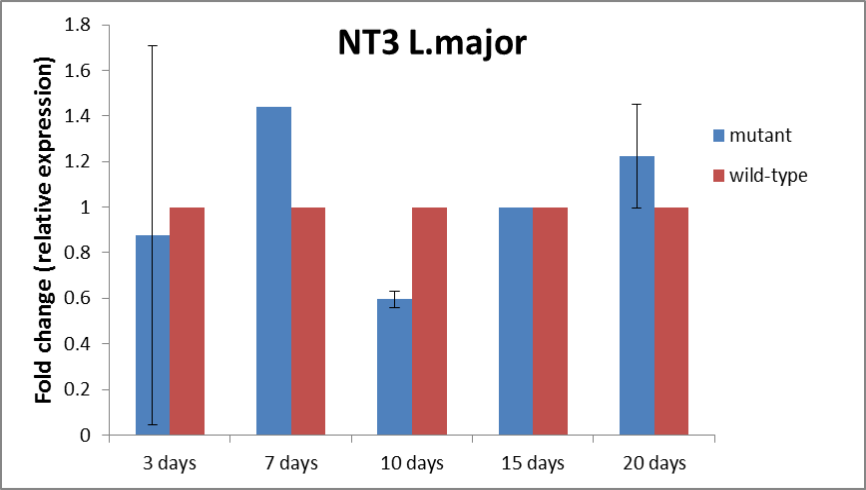
NT3 gene expression in mutant *Leishmania major* on 3, 7, 10, 15 and 20 d post electroporation. Real time-RT PCR results analysis revealed that NT3 gene expression in transgenic *Leishmania* was at 3 d: 0.875±0.832201825 (*P*<0.85), 7 day: 1.439±0.035264924 (*P*<0.003), 10 d: 0.596±0.228100628 (*P*<0.01), 15 d: 1.00±1.73422E-15 (*P*<0.39), and 20 d: 1.225±1.095573406 (*P*<0.79)

Real time-RT PCR results analysis on days 3, 7, 10, 15 and 20 after transfection revealed that NT3 gene expression in transgenic *Leish-mania* was reduced on the tenth day, but it had upward trend up to the twentieth day. There was a significant difference in increased expression of this gene on day 7 and reduction of its expression on tenth day in mutant parasite (*P*<0.05) (Fig. 1).

### NT3 protein identity by Western blotting

**Fig. 2:**
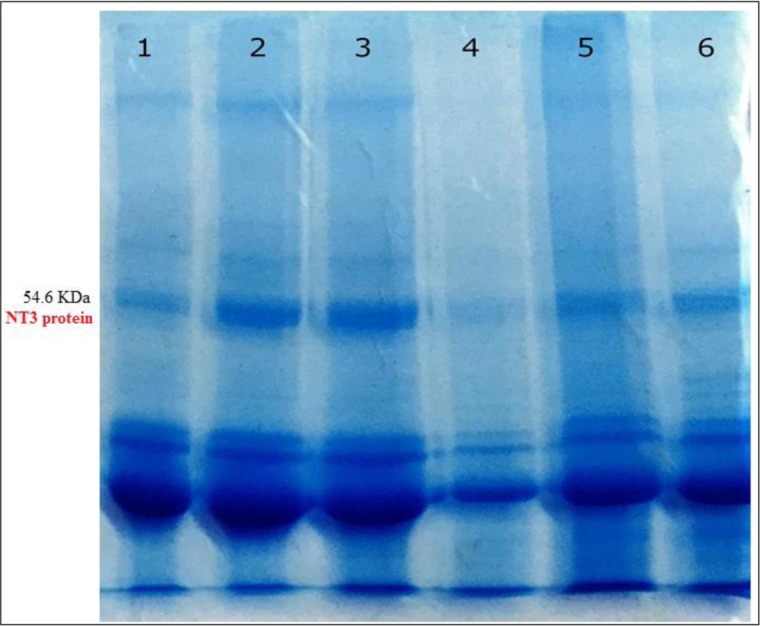
**SDS-PAGE of NT3 gene expression in transgenic *Leishmania* on 3, 7, 10, 15 and 20 days after transfection.** Wild-type as positive control (lane1), mutant strain in 20th (lane2), 15th (lane3), 10th (lane4), 7th (lane5), 3th (lane6) days after electroporation

**Fig. 3:**
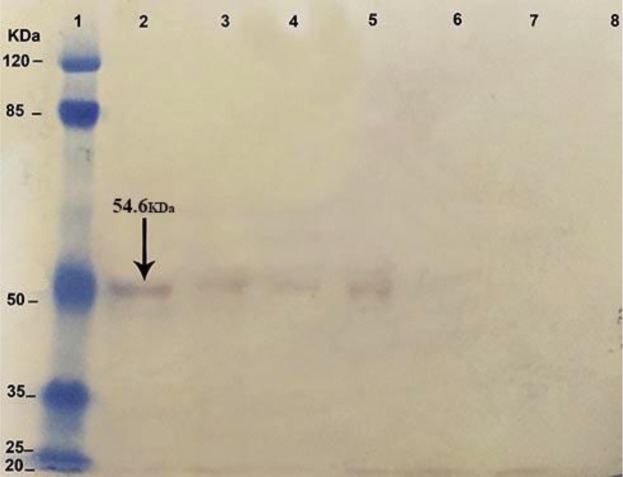
Western blot analysis of NT3 gene expression in transgenic *Leishmania* on 3, 7, 10, 15 and 20 d after transfection. This result confirmed NT3 protein expression in the previous test. Total protein extracted from 1×10^6^ cells of wild-type promastigotes and transgenic promastigotes were electrophoresed and subjected to western blot analysis with anti NT3 Ab. NT3 protein is 54.6 KDa. Protein Marker (lane1), wild-type as positive control (lane2), mutant strain in 20^th^ (lane3), 15^th^ (lane4), 10^th^ (lane5), 7^th^ (lane6), 3^th^ (lane7) d after electroporation and NT3 construct as negative control (lane8). The result of this experiment shows that NT3 gene in transgenic *Leishmania* was expressed and the expression was too low

NT3 gene in transgenic *Leishmania* was expressed on 20, 15, and 10 d (Fig 2) and its expression was too low (Fig. 3).

### Macrophages infection

**Fig. 4:**
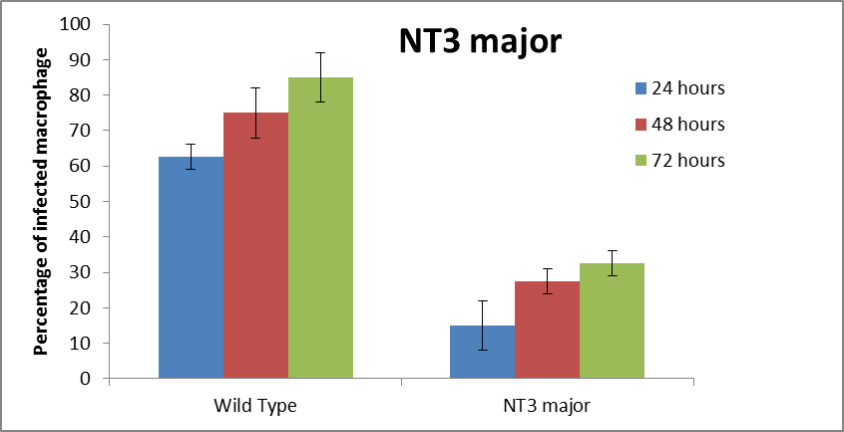
Percent of infective macrophage in contact with the mutant type with NT3 *Leishmania major* and wild type. The percentages of infected macrophages in the mutant strains was 15 ± 7.07% (n=2), 27.5± 3.5% (n=2) and 32.5 ± 3.5% (n=2) at 24, 48 and 72 h, respectively. In contrast, the percentage of infected macrophages in the wild-type was 62.5± 3.5% (n=2), 75 ± 7.07% (n=2, and 85 ± 7.07% (n=2) at 24, 48 and 72 h, respectively

The percentages of infected macrophages in contact with the mutant *L. major* and wild-type strain were evaluated after 24, 48 and 72 h. The infected macrophages with the mutant parasites at the three-time points were lower than that of the wild-type strain. The statistical analysis showed a significant difference (*P*<0.05) between percentages of the infected macrophages in the mutant *L. major* and the wild-type strains (Fig. 4).

**Fig. 5:**
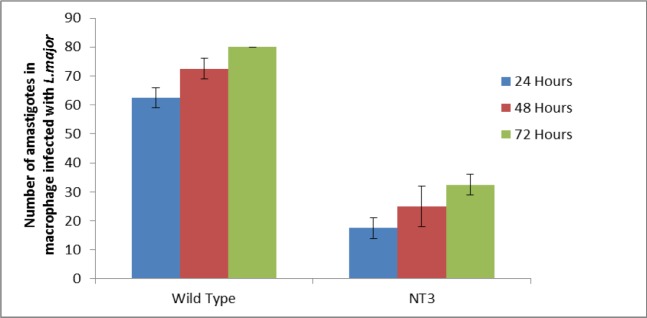
The number of amastigotes in macrophages infected with transgenic Leishmania *major*. The number of amastigotes in macrophages infected with transgenic *L. major* was 17.5±3.5 (n=2), 25±7.07 (n=2), and 32.5±3.5 (n=2) in 24, 48 and 72 h, respectively; on the other hand, the number of amastigotes in the wild-type was 62.5±3.5 (n=2) in 24 h, 72.5±3.5 (n=2) in 48 h, and 80±0 (n=2) in 72 h

The number of amastigotes infected macrophages with transgenic *L. major* was lower than that of the wild-type strain. The statistical analysis showed a significant difference (*P*<0.05) between the number of amastigotes infected macrophages in the mutant *L. major* and the wild-type strains (Fig. 5). In the light microscopy analysis, no difference was found in the shape and size (by eyepiece micrometer) of amastigotes in the mutant parasite and wild-type strain.

**Fig. 6:**
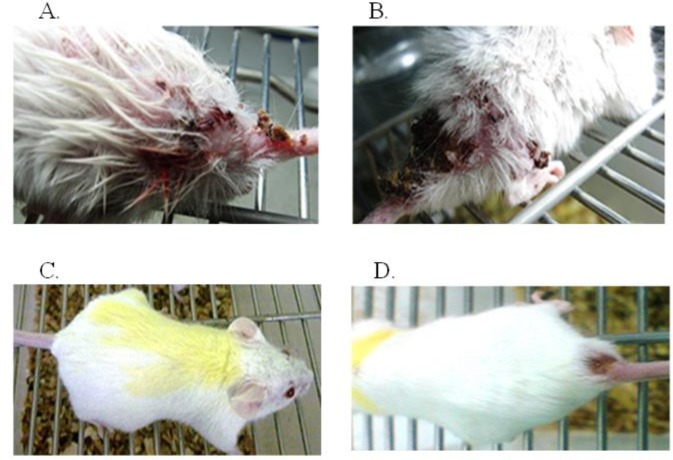
Compare BALB/c mice infected lesions in wild type and transgenic parasites. The lesions were created within 30 d in the mice injected with wild type (B) and were not created within 30 d in the mice injected with mutant parasites (A). It was created during 70 d in the mice infected with mutant parasites (C, D lesions70 d in the mice infected with wild type). Many lesions were seen with a diameter of more than 5 mm in the mice infected with wild type. While, one lesion with a diameter of less than 5 mm was observed in the mice infected with the NT3 mutant strain

The lesions in BALB/c mice, created within 30 d in the mice infected with the wild type (Fig. 6 A) but in the mice infected with mutant parasites any lesions were not created within 30 d (Fig. 6 B). It was created within 70 d in the mice infected with mutant parasites (Fig. 6 C,D within 70 d in the mice infected with the wild type). More than one ulcer with a diameter greater than 5 mm were found in the mice receiving the wild type, while one ulcer with a diameter of less than 5 mm was observed in the mice infected with the mutant strain.

## Discussion

In *L. major*, NT3 gene is the essential transporter for purine nucleobases and it has many roles in the biology of *L. major* (14). In this study, antisense RNA was used to down-regulating NT3 gene expression. Antisense RNA usually creates a barrier to the transmission of genetic information from RNA to proteins (23). In this research, expression of this gene was evaluated after transfection with mutant *Leishmania* in vitro and in vivo.

Antisense has been effective on the expression of this gene since the tenth day. Although on the seventh day, there was a significant increase in the expression of the gene (*P*<0.05), on the tenth day, the antisense had a significant decreasing on the expression of the gene (*P*<0.05). Since the proliferation of promastigotes is performed on every 7 h, the anti-sense effect is temporary so, parasites lose plasmid and normally express this gene. Consequently, the gene expression from the tenth day shows an increasing trend.

NT3 protein was down-regulated. Nevertheless, the NT3 protein with a molecular weight of 54.6 kDa was produced followed by gene expression. We evaluated the percentage of infected macrophage in murine macrophages J774 within 24, 48 and 72 h after electroporation of transgenic *Leishmania* promastigotes compared with wild-type parasites of *L. major* promastigotes. There is the reduction of the infected macrophages and reduction in the number of amastigotes inside the infected macrophages with mutant *Leishmania.* On the other hand, in the infected macrophages with mutant *Leishmania,* there is reduced the ability of proliferation of parasites or virulence of parasites. Macrophages stimulate the cellular response and eventually eliminate amastigotes inside the macrophages (24, 25). In this study, size and number of ulcers in BALB/c mice were investigated in vivo. Size and number of ulcers in BALB/c mice infected with transgenic *Leishmania* promastigotes were less than the BALB/c mice infected with wild-type parasites. However, the incubation period of ulcers in BALB/c mice infected with transgenic *Leishmania* promastigotes was longer than the BALB/c mice infected with wild-type parasites. In this case, our results are agreement with the research of Mottram (26). Our results showed the reduction of the expression the gene and the virulence of parasites may be caused by antisense RNA.

## Conclusion

We were able to temporarily downregulate gene expression of NT3. Further studies are required to provide new targets for interventional therapy in *Leishmania* infection.
